# Tracing the Trail of Protons through Complex I of the Mitochondrial Respiratory Chain

**DOI:** 10.1371/journal.pbio.1001129

**Published:** 2011-08-23

**Authors:** Arnaud Mourier, Nils-Göran Larsson

**Affiliations:** Max-Planck-Institut für Biologie des Alterns, Cologne, Germany

## Abstract

Mitochondria are the structures that produce the bulk part of the cellular energy currency ATP, which drives numerous energy requiring processes in the cell. This process involves a series of large enzyme complexes—the respiratory chain—that couples the transfer of electrons to the creation of a concentration gradient of protons across the inner mitochondrial membrane, which drives ATP synthesis. Complex I (or NADH-quinone oxidoreductase) is the largest and by far the most complicated of the respiratory chain enzyme complexes. The molecular mechanism whereby it couples electron transfer to proton extrusion has remained mysterious until very recently. Low-resolution X-ray structures of complex I have, surprisingly, suggested that electron transfer in the hydrophilic arm, protruding into the mitochondrial matrix, causes movement of a coupling rod that influences three putative proton pumps within the hydrophobic arm embedded in the inner mitochondrial membrane. In this Primer, we will briefly introduce the recent progress made in this area and highlight the road ahead that likely will unravel the detailed molecular mechanisms of complex I function.

Mammalian cells generate most of their ATP by a process called oxidative phosphorylation located in the mitochondrial inner membrane (Figure 1). Two functional entities carry out this process: first, the respiratory chain, historically defined as four complexes (I, II, III, and IV) that transfer electrons to molecular oxygen via electron carriers such as quinone and cytochrome *c*, and second, the system that phosphorylates ADP to produce ATP, which comprises the ATP synthase and some inner membrane transporters such as the ATP/ADP translocase (ANT) and the phosphate carrier (Pic).

**Figure 1:**
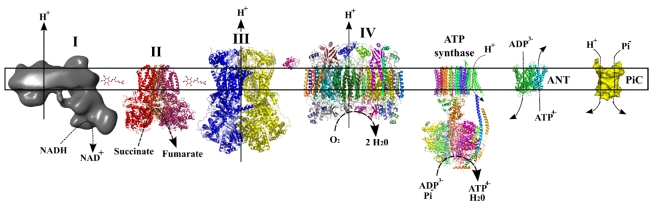
Schematic depiction of the oxidative phosphorylation system [**28**]. In this scheme the structural models are not drawn to scale.

In 1925 Otto Warburg first described the phenomenon of cellular respiration [1],[2], and 15 years later Herman Kalckar showed that respiration was linked to ATP production [3]. The mechanism coupling respiration to ATP synthesis remained enigmatic, however, until Peter Mitchell formulated the chemiosmotic theory in 1961 [4]. According to this theory, the electron transfer by the respiratory chain complexes leads to vectorial extrusion of protons from the matrix into the intermembrane space of mitochondria. The low proton conductance of the inner mitochondrial membrane allows protons to accumulate outside the matrix space forming an osmotic force (also defined as the proton motive force or Δp). The re-entry of protons into the matrix space via the ATP synthase, located in the inner membrane, induces conformational changes that catalyze the synthesis of ATP from ADP and P_i_.

Three of the respiratory chain complexes translocate protons as they perform electron transfer: NADH–ubiquinone oxidoreductase (complex I), ubiquinol–cytochrome *c* oxidoreductase (complex III), and cytochrome *c* oxidase (complex IV). Complex I is the first and largest of the respiratory chain enzyme complexes; it is directly involved in maintaining cellular reduction–oxidation (redox; NADH/NAD^+^) homeostasis and indirectly involved, by its electron transfer and proton extrusion activity, in ATP production. Complex I is also known to be a major source of reactive oxygen species (ROS) [5],[6], which are implicated in cell signaling, disease, and aging in mammals [7]. The mammalian complex I has a molecular mass of about 1.0 MDa and is composed of at least 45 different subunits, seven of which are encoded by the mitochondrial (mt)DNA. Mutations in complex I subunits have been associated with a range of pathologies with poorly understood pathophysiology. Patients with complex I deficiency have a wide variety of phenotypes ranging from neonatal onset of fatal forms of lactic acidosis, cardiomyopathy, and neurodegeneration (e.g., infantile bilateral striatal necrosis, Leigh syndrome) to adult onset of optic atrophy without major involvement of other organs (e.g., Leber's hereditary optic neuropathy) [8]. Whereas important progress has been made in the diagnosis and genetic analysis of complex I deficiency, the link between the molecular defect and the observed symptoms is hardly understood at all. Specific mutations of complex I subunit genes in the nucleus or mtDNA will likely result in distinct molecular phenotypes with different impacts on ATP production, redox status, ROS production, and possibly also other important processes. Despite more than 50 years of study and speculation, complex I has remained the least well-understood component of the respiratory chain; an in-depth understanding of this molecular machinery is not only of basic scientific interest but will also help to explain human disease phenotypes.

Initial analyses of complex I structure in different organisms by electron microscopy revealed an unusual L-shaped form with a soluble arm extending into the mitochondrial matrix and a perpendicular arm buried in the inner mitochondrial membrane (Figure 1) [9]–[11]. The matrix part contains one flavin and eight or nine iron-sulfur-clusters, necessary for electron transfer from NADH to ubiquinone (Figure 2A). This electron path was first proposed based on results from electron paramagnetic resonance studies and later confirmed by a high-resolution X-ray crystallographic structure of the hydrophilic domain of complex I from the bacterium *Thermus thermophilus* [12]. Analysis of the atomic structure allowed definition of the path for flow of electrons through the matrix arm and even revealed structural elements that could undergo conformational changes during electron transfer through complex I; however, the proton pathway and the mechanism linking electron transfer to proton translocation remained unresolved until very recently. The simultaneous reports of low-resolution X-ray structures of the entire complex I from *T. thermophilus* and the yeast *Yarrowia lipolytica* led to a breakthrough in our understanding of how electron transfer is linked to proton transport [13],[14]. In both the prokaryotic and eukaryotic complex I structure, the integral membrane proteins contain three large subunits that are structurally homologous to bacterial sodium–proton antiporters. Interestingly, the structures also revealed the presence of a long helix running parallel to the membrane and in direct contact with the key functional parts of the three putative proton pumps. This unexpected finding led to the proposal that this helix might act as a coupling rod that transmits conformational changes induced by electron transport to the proton pumps located quite far away in the more than 180 Å–long membrane arm (Figure 2A).

**Figure 2:**
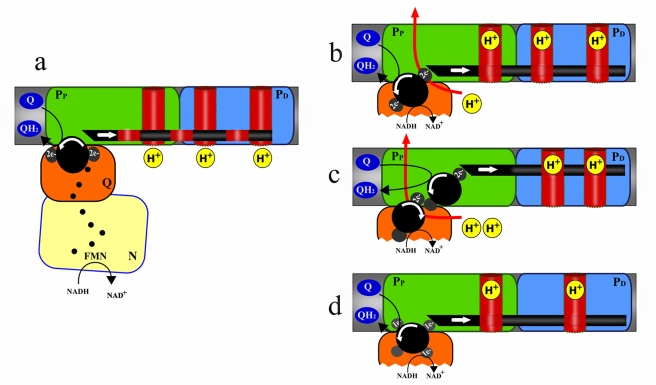
Schematic model of complex I with four functional modules as proposed by Hunte et al. [**14**]. (a) Representation of the rod model of coupling between electron transfer and proton extrusion. (b) The proton pathways according to the chimera model proposed by Friedrich and Sazanov [13],[15]. (c) Scheme representing the model proposed by Ohnishi and Salerno [16],[17]. (d) Representation of the indirect model recently proposed by Brandt [20]. P_p_ indicates the proximal proton pumping domain.

Despite a general agreement that four protons are translocated per pair of electrons transferred, no consensus model of proton pathways has been developed. The controversy is at least partly explained by the realization that the homology between Na^+^/H^+^ antiporters and the three putative pumps does not necessarily mean that all of these are functional proton pumps in complex I. Three models have been proposed based on the number of protons transferred as a direct consequence of the catalytic reduction of quinone (direct transfer) or by proton translocating pumps (indirect transfer). The “chimera model” proposed by Friedrich [15] and Sazanov [13] involves both direct and indirect proton transfer. They based their model on the suggestion from structural analyses that all three proton pumps are functional and translocate one proton each by indirect transfer, with the fourth proton being translocated by direct transfer during electron transfer to quinone (Figure 2B). Ohnishi and Salerno [16] also proposed a combined mechanism involving a second quinone-binding site and two functional proton pumps (Figure 2C). This model is based on the proposed existence of two distinct quinone-binding sites, with one of them involved in the direct transfer of two protons [16],[17]. The third model proposed by the groups of Yagi [18], Verkhovskaya [19], and Brandt [20] argues that all protons are translocated by an indirect mechanism (Figure 2D). A refined version of this third model [20] proposes that transmembrane proton translocation involves only two proton pumps and that each transferred electron induces one coupling-rod-induced conformational change, causing the extrusion of two protons (Figure 2D). Consequently, the transfer of two electrons to quinone leads to two cycles of conformational changes, which in total leads to the extrusion of four protons. Recent studies in *E. coli* underscore the functional importance of the putative proton pumps located in the distal proton pumping domain (P_D_) of the membrane arm [21]–[23]; however, the structural interconnection of the three putative proton pumps makes it very difficult to assess the individual contribution of each pump in the proton extrusion process.

The Brandt group has used *Y. lipolytica* as a powerful model system to study structure and proton translocation of complex I. Loss of complex I in this yeast normally results in cell lethality, but this problem can be overcome by expressing an alternative single subunit NADH:ubiquinone oxidoreductase in the mitochondrial matrix [24]. In this issue of *PLoS Biology*, Brandt and colleagues report that deletion of a small complex I subunit, NB8M (NDUFB7 in human complex I), results in formation of a stable subcomplex of complex I [25]. By using three independent approaches, the authors elegantly demonstrate that the P_D_ module is missing in these mutants. Unexpectedly, the partially assembled complex is still active and can translocate protons. In a series of clever experiments, the authors characterize this mutant to investigate the contribution of the different parts of the membrane arm of complex I in proton translocation. The mutated complex shows a reduced electron-to-proton stoichiometry and a detailed characterization shows that half of the protons translocated by the wild-type complex I are indeed translocated by the P_D_ module by an indirect process. This study provides the first functional characterization of the two pumping modules in eukaryotic complex I. Intriguingly, the P_D_ module is located well over 100 Å away from the closest redox centre, which provides experimental support for the proposed coupling-rod-dependent mechanism to trigger proton translocation. In addition, the authors show that the P_D_ module of complex I contains at least one active proton pump. It should be noted, however, that the decrease in stoichiometry from four to two protons extruded per two electrons translocated when the P_D_ module is absent is also compatible with other proposed models (Figure 2). The study by Brandt and coworkers is an important step towards understanding the molecular link between electron transfer and proton translocation by complex I; however, many important questions remain to be answered. For example, what are the other sites for proton pumping? What is the driving force for movement of the coupling rod? A detailed understanding of the quinone chemistry inside complex I will be necessary to reveal if there is a direct coupling mechanism and to clarify the origin of the force driving the rod. High-resolution structures of complex I in various active conformations will be important goals for the future. The *Y. lipolytica* system is very powerful as it contains a similar number of subunits as human complex I and, in addition, is accessible to genetics by manipulation of nuclear genes. A complicating factor in genetic analysis is that the putative proton pumping subunits are encoded by mtDNA and dissection of their function will therefore necessitate the development of mtDNA-transformation protocols in *Y. lipolytica*, which may be based on existing methods in *Saccharomyces cerevisiae* [26],[27]. The golden era for complex I research has just arrived and we can anticipate many exciting insights in future into the function of this molecular gadget.

## Abbreviations

mtDNA: mitochondrial DNA; P_D_, distal proton pumping domain
